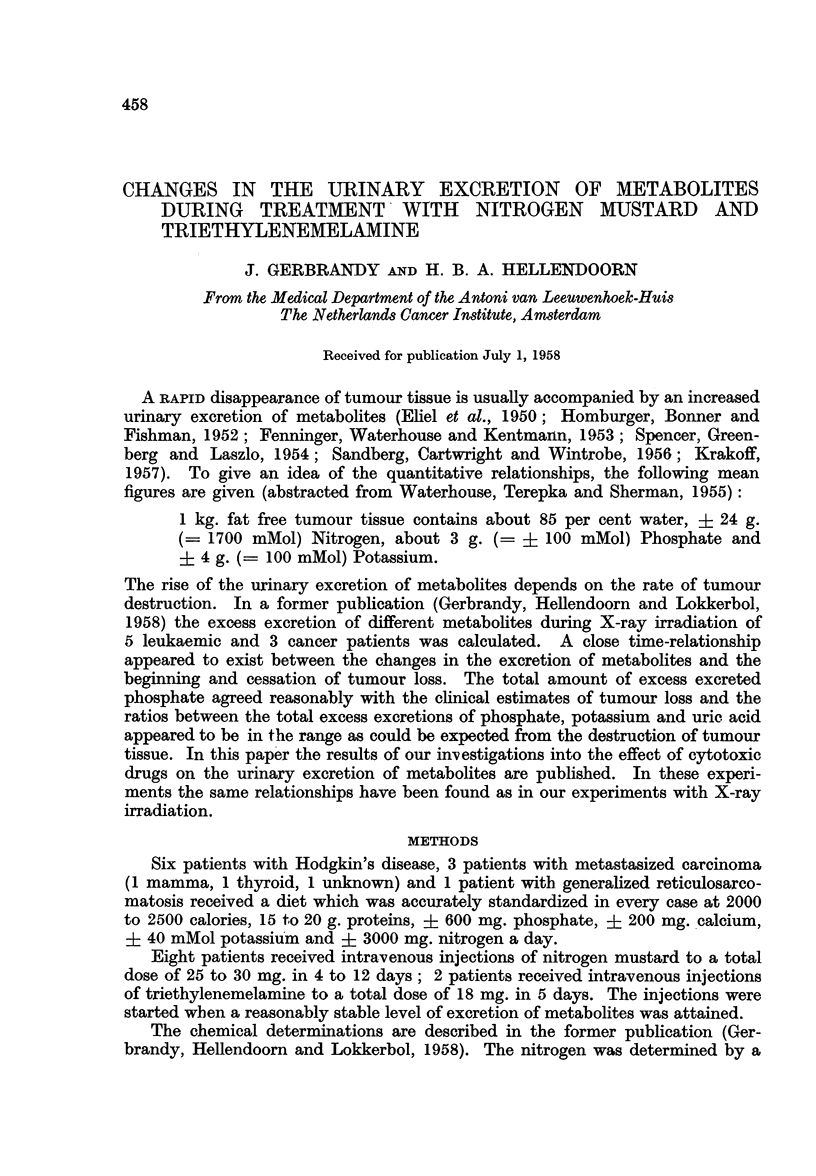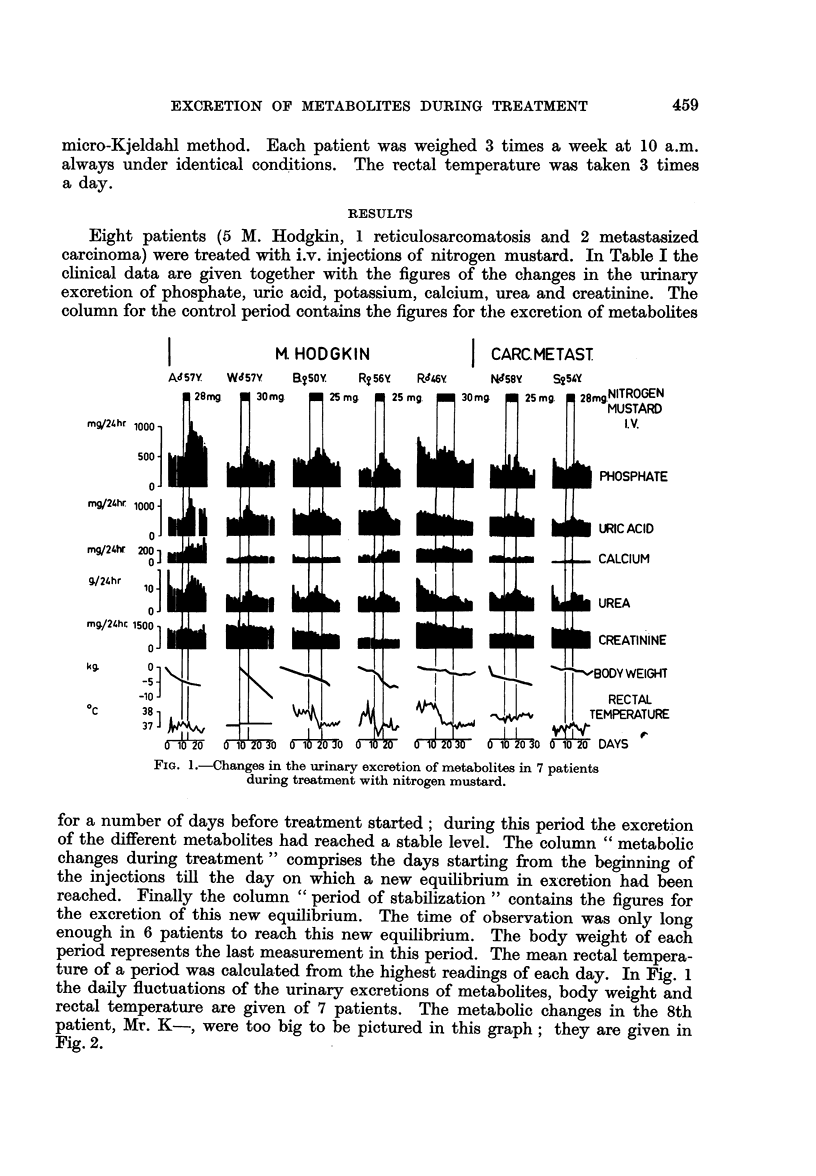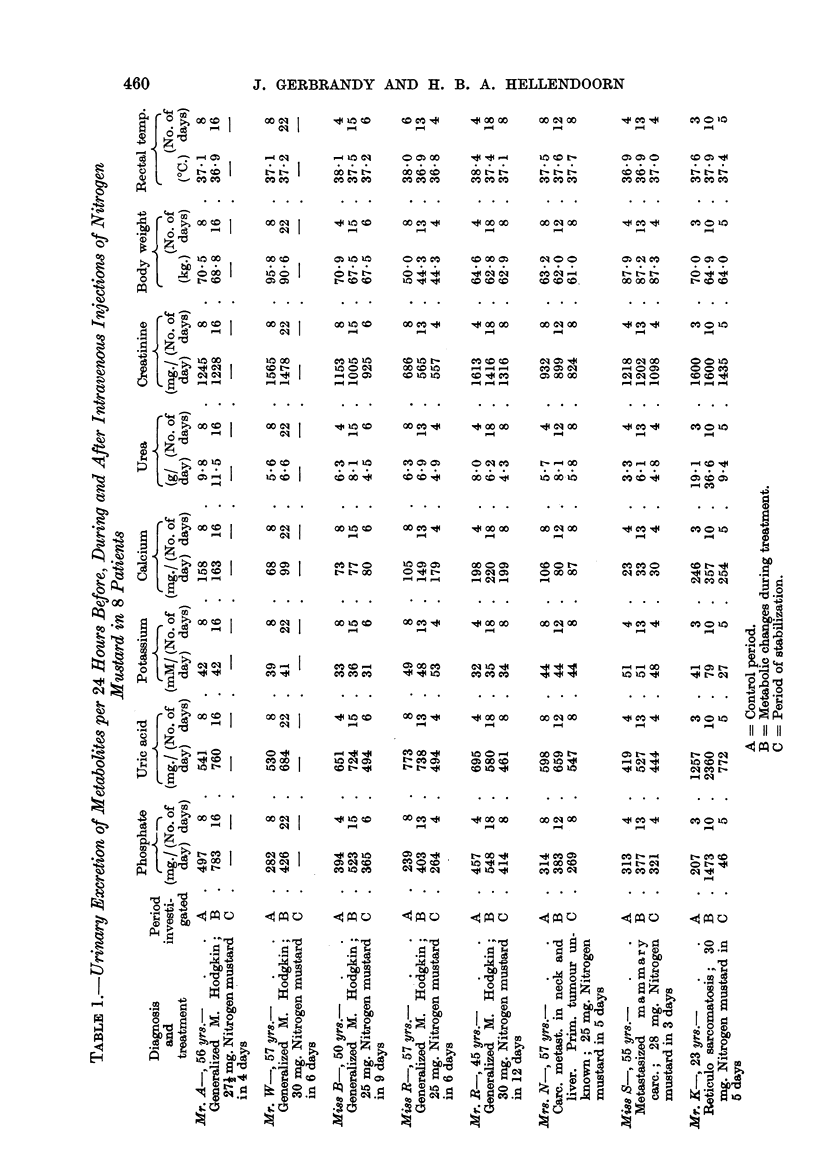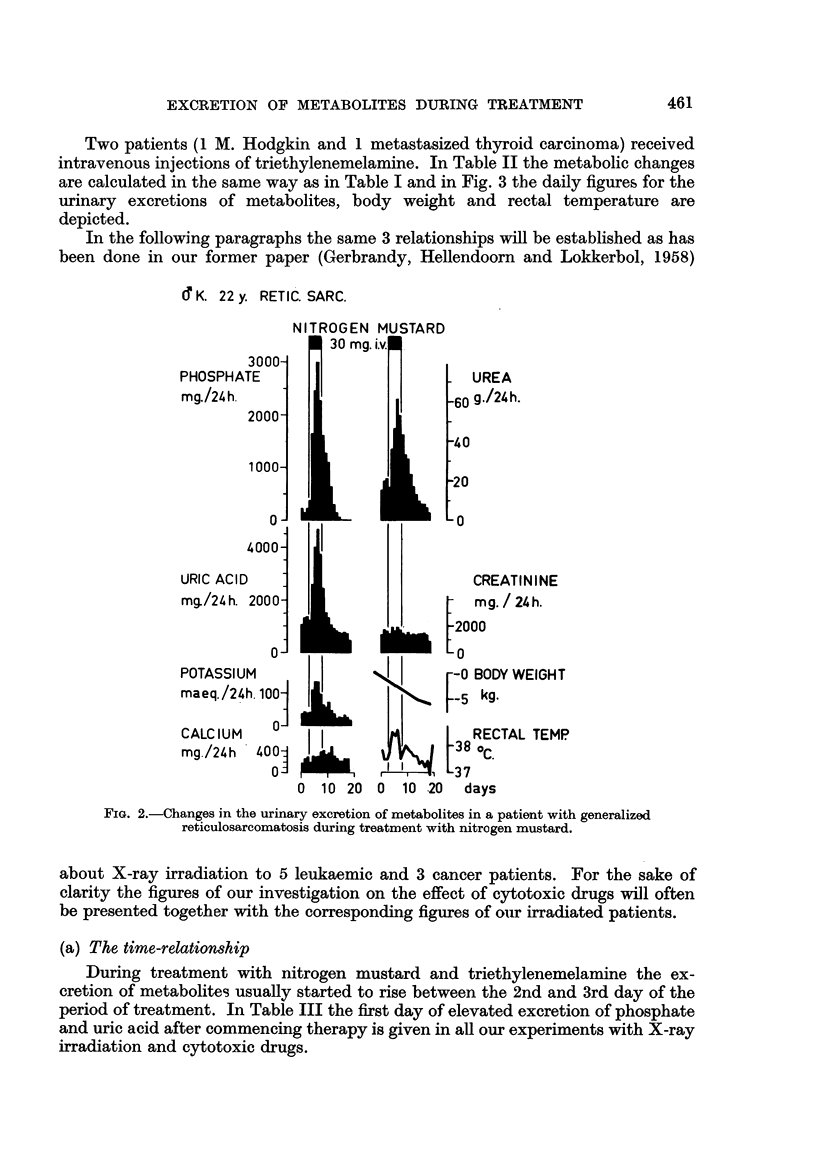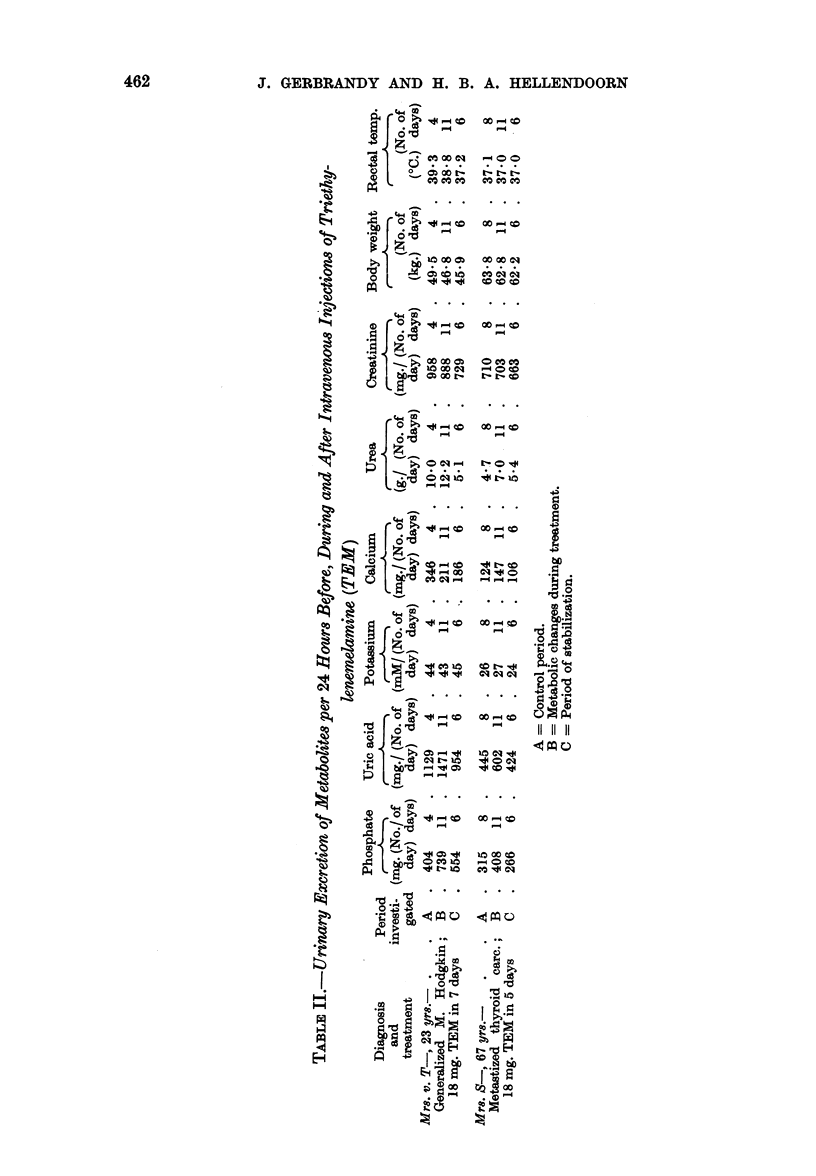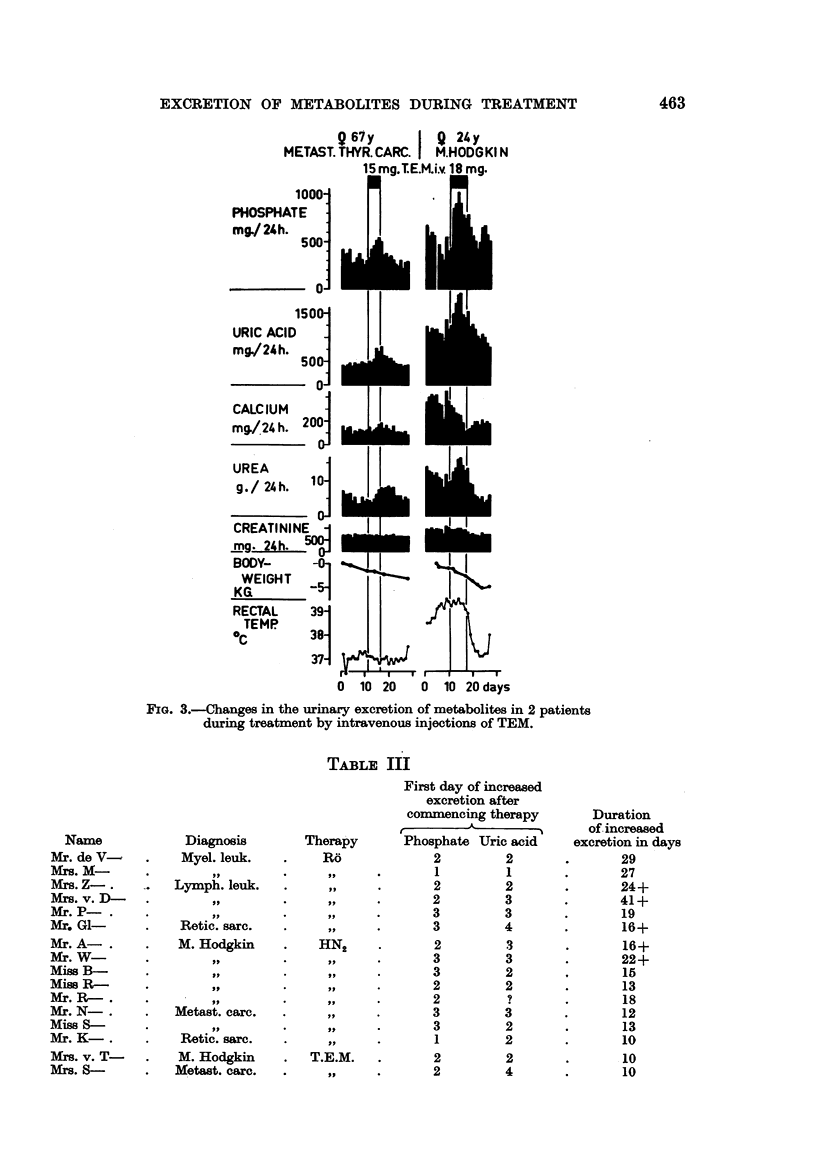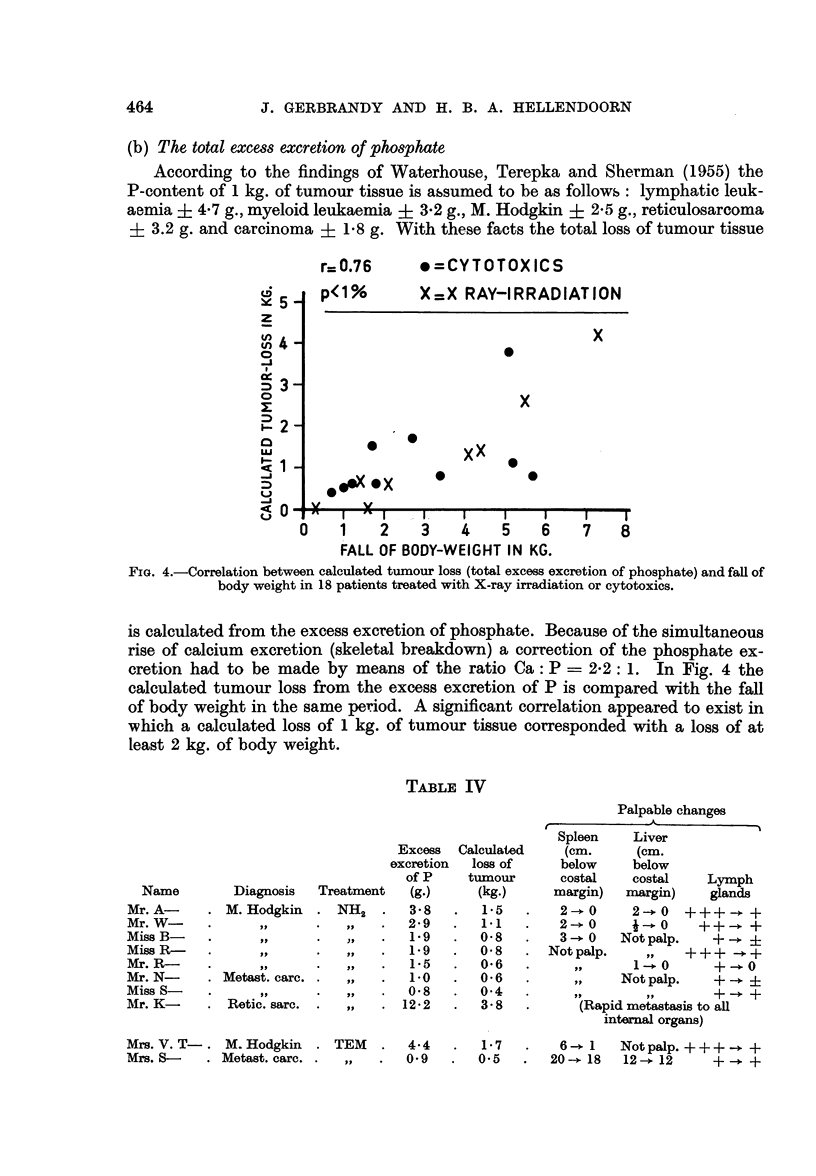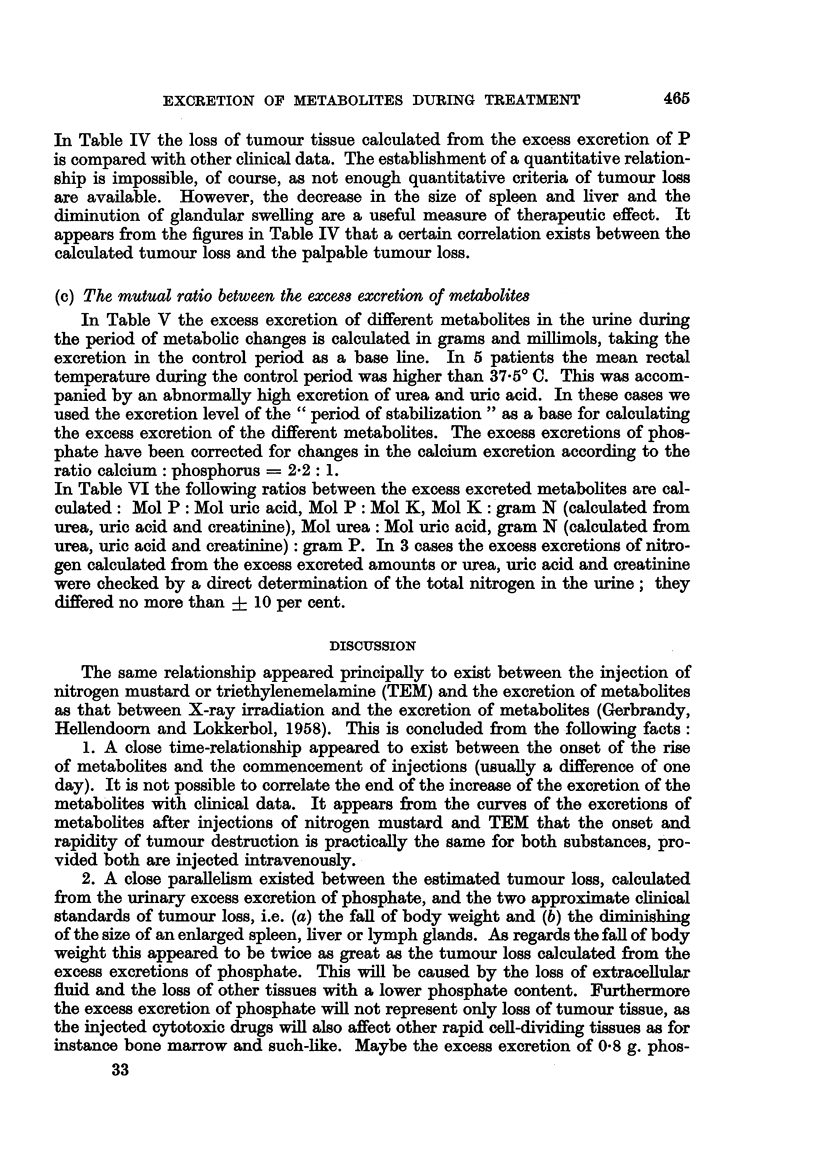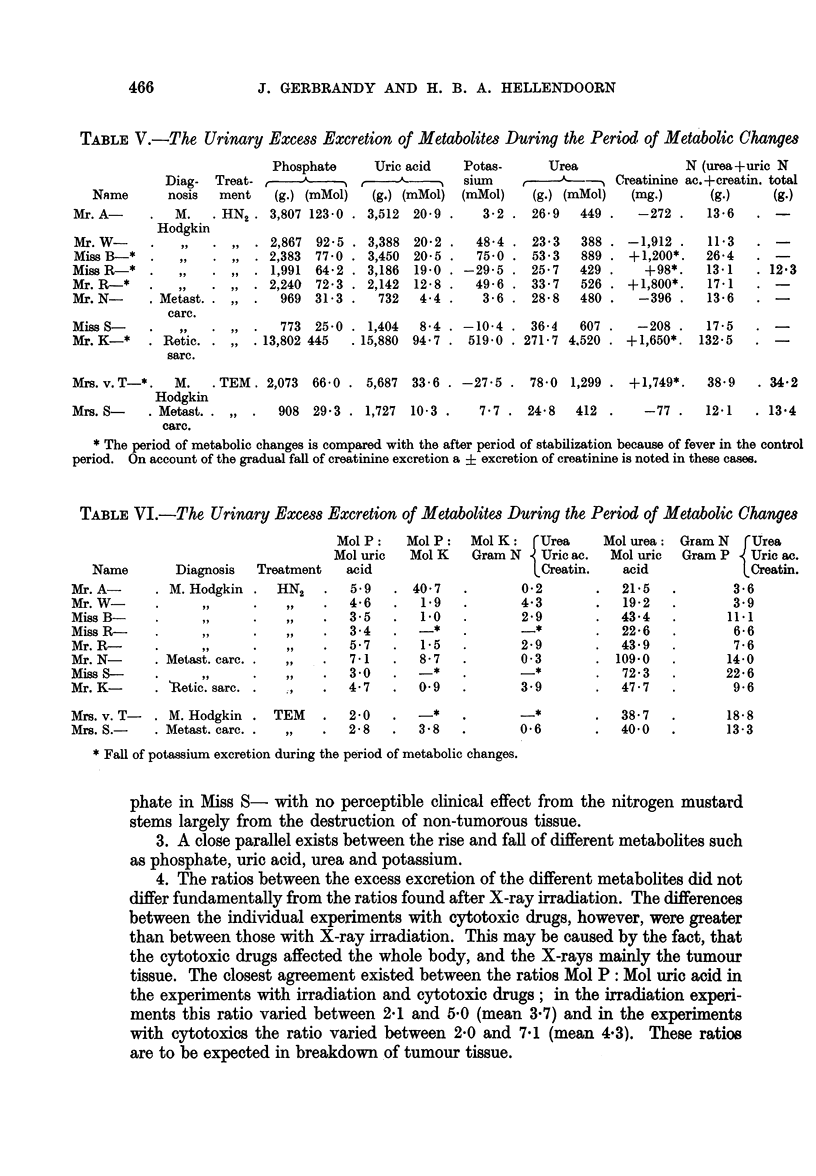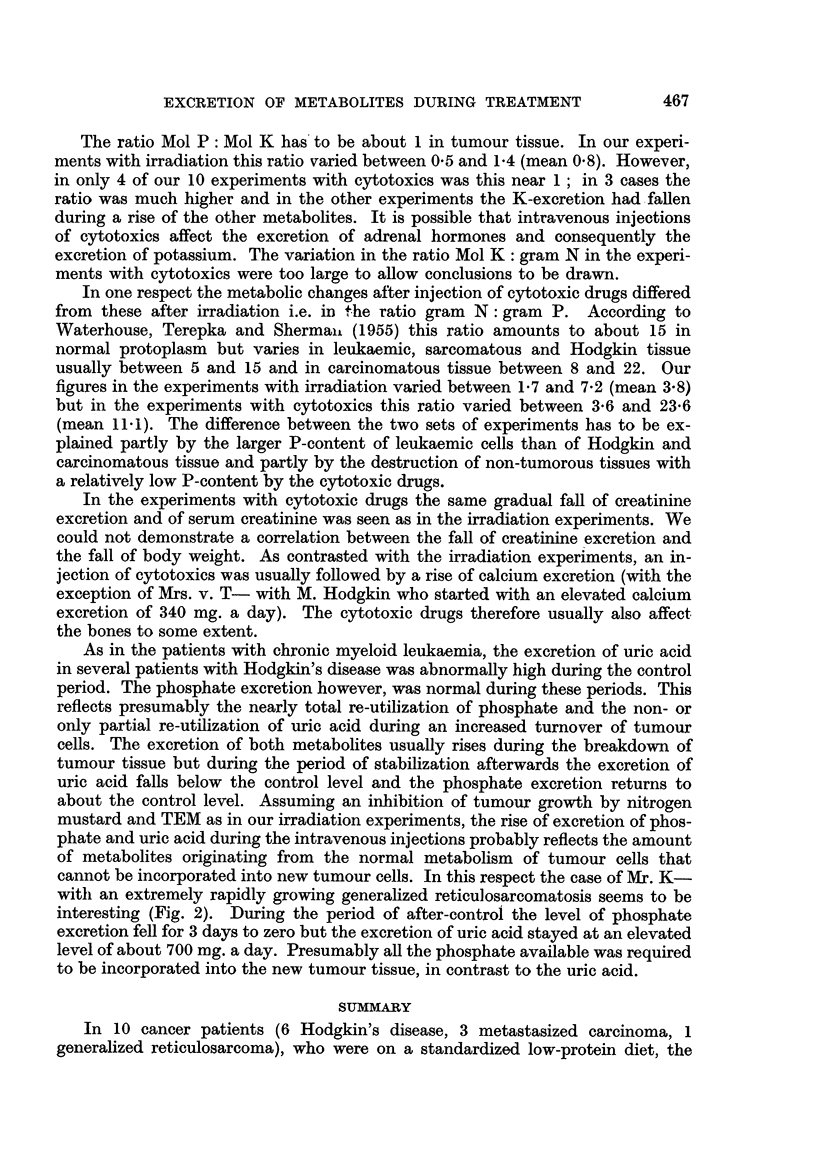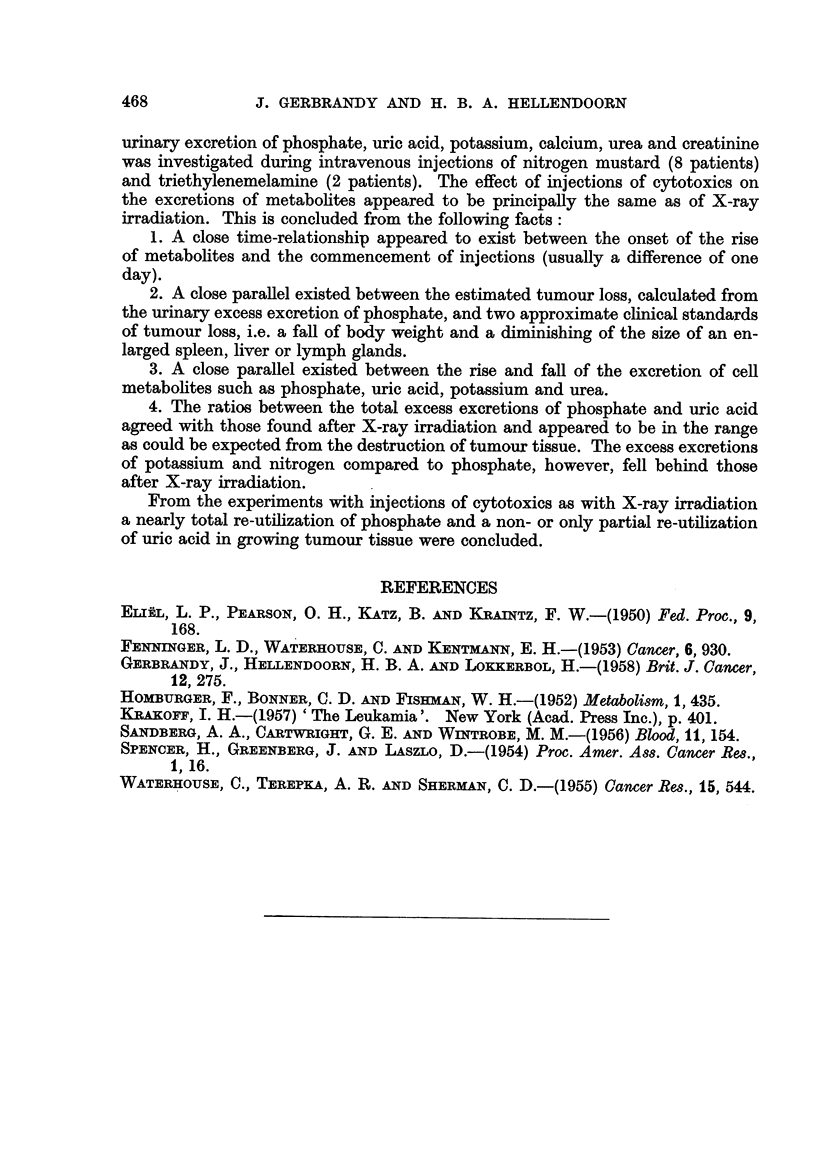# Changes in the Urinary Excretion of Metabolites during Treatment with Nitrogen Mustard and Triethylenemelamine

**DOI:** 10.1038/bjc.1958.54

**Published:** 1958-09

**Authors:** J. Gerbrandy, H. B. A. Hellendoorn


### CHANGES IN THE URINARY EXCRETION OF METABOLITES DURING TREATMENT WITH NITROGEN MUSTARD AND TRIETHYLENEMELAMINE

J. GERBRANDY AND H. B. A. HELLENDOORN

*From the Medical Department of the Antoni van Leeuwenhoek-Huis*
*The Netherlands Cancer Institute, Amsterdam*

Received for publication July 1, 1958

A RAPID disappearance of tumour tissue is usually accompanied by an increased urinary excretion of metabolites (Eliel *et al.*, 1950 ; Homburger, Bonner and Fishman, 1952 ; Fenninger, Waterhouse and Kentmann, 1953 ; Spencer, Greenberg and Laszlo, 1954 ; Sandberg, Cartwright and Wintrobe, 1956 ; Krakoff, 1957). To give an idea of the quantitative relationships, the following mean figures are given (abstracted from Waterhouse, Terepka and Sherman, 1955) :

> 1 kg. fat free tumour tissue contains about 85 per cent water, $\pm$ 24 g. (= 1700 mMol) Nitrogen, about 3 g. (= $\pm$ 100 mMol) Phosphate and $\pm$ 4 g. (= 100 mMol) Potassium.

The rise of the urinary excretion of metabolites depends on the rate of tumour destruction. In a former publication (Gerbrandy, Hellendoorn and Lokkerbol, 1958) the excess excretion of different metabolites during X-ray irradiation of 5 leukaemic and 3 cancer patients was calculated. A close time-relationship appeared to exist between the changes in the excretion of metabolites and the beginning and cessation of tumour loss. The total amount of excess excreted phosphate agreed reasonably with the clinical estimates of tumour loss and the ratios between the total excess excretions of phosphate, potassium and uric acid appeared to be in the range as could be expected from the destruction of tumour tissue. In this paper the results of our investigations into the effect of cytotoxic drugs on the urinary excretion of metabolites are published. In these experiments the same relationships have been found as in our experiments with X-ray irradiation.

### METHODS

Six patients with Hodgkin's disease, 3 patients with metastasized carcinoma (1 mamma, 1 thyroid, 1 unknown) and 1 patient with generalized reticulosarcomatosis received a diet which was accurately standardized in every case at 2000 to 2500 calories, 15 to 20 g. proteins, $\pm$ 600 mg. phosphate, $\pm$ 200 mg. calcium, $\pm$ 40 mMol potassium and $\pm$ 3000 mg. nitrogen a day.

Eight patients received intravenous injections of nitrogen mustard to a total dose of 25 to 30 mg. in 4 to 12 days ; 2 patients received intravenous injections of triethylenemelamine to a total dose of 18 mg. in 5 days. The injections were started when a reasonably stable level of excretion of metabolites was attained.

The chemical determinations are described in the former publication (Gerbrandy, Hellendoorn and Lokkerbol, 1958). The nitrogen was determined by a micro-Kjeldahl method. Each patient was weighed 3 times a week at 10 a.m. always under identical conditions. The rectal temperature was taken 3 times a day.

### RESULTS

Eight patients (5 M. Hodgkin, 1 reticulosarcomatosis and 2 metastasized carcinoma) were treated with i.v. injections of nitrogen mustard. In Table I the clinical data are given together with the figures of the changes in the urinary excretion of phosphate, uric acid, potassium, calcium, urea and creatinine. The column for the control period contains the figures for the excretion of metabolites

FIG. 1.—Changes in the urinary excretion of metabolites in 7 patients during treatment with nitrogen mustard.

for a number of days before treatment started ; during this period the excretion of the different metabolites had reached a stable level. The column " metabolic changes during treatment " comprises the days starting from the beginning of the injections till the day on which a new equilibrium in excretion had been reached. Finally the column " period of stabilization " contains the figures for the excretion of this new equilibrium. The time of observation was only long enough in 6 patients to reach this new equilibrium. The body weight of each period represents the last measurement in this period. The mean rectal temperature of a period was calculated from the highest readings of each day. In Fig. 1 the daily fluctuations of the urinary excretions of metabolites, body weight and rectal temperature are given of 7 patients. The metabolic changes in the 8th patient, Mr. K—, were too big to be pictured in this graph ; they are given in Fig. 2.

TABLE 1.—*Urinary Excretion of Metabolites per 24 Hours Before, During and After Intravenous Injections of Nitrogen Mustard in 8 Patients*

| Diagnosis and treatment | Period investigated | Phosphate (mg./day) | Phosphate (No. of days) | Uric acid (mg./day) | Uric acid (No. of days) | Potassium (mM/day) | Potassium (No. of days) | Calcium (mg./day) | Calcium (No. of days) | Urea (g/day) | Urea (No. of days) | Creatinine (mg./day) | Creatinine (No. of days) | Body weight (kg.) | Body weight (No. of days) | Rectal temp. (°C.) | Rectal temp. (No. of days) |
|---|---|---|---|---|---|---|---|---|---|---|---|---|---|---|---|---|---|
| *Mr. A—, 56 yrs.*— Generalized M. Hodgkin; 27½ mg. Nitrogen mustard in 4 days | A | 497 | 8 | 541 | 8 | 42 | 8 | 158 | 8 | 9·8 | 8 | 1245 | 8 | 70·5 | 8 | 37·1 | 8 |
| | B | 783 | 16 | 760 | 16 | 42 | 16 | 163 | 16 | 11·5 | 16 | 1228 | 16 | 68·8 | 16 | 36·9 | 16 |
| | C | — | — | — | — | — | — | — | — | — | — | — | — | — | — | — | — |
| *Mr. W—, 57 yrs.*— Generalized M. Hodgkin; 30 mg. Nitrogen mustard in 6 days | A | 282 | 8 | 530 | 8 | 39 | 8 | 68 | 8 | 5·6 | 8 | 1565 | 8 | 95·8 | 8 | 37·1 | 8 |
| | B | 426 | 22 | 684 | 22 | 41 | 22 | 99 | 22 | 6·6 | 22 | 1478 | 22 | 90·6 | 22 | 37·2 | 22 |
| | C | — | — | — | — | — | — | — | — | — | — | — | — | — | — | — | — |
| *Miss B—, 50 yrs.*— Generalized M. Hodgkin; 25 mg. Nitrogen mustard in 9 days | A | 394 | 4 | 651 | 4 | 33 | 8 | 73 | 8 | 6·3 | 4 | 1153 | 8 | 70·9 | 4 | 38·1 | 4 |
| | B | 523 | 15 | 724 | 15 | 36 | 15 | 77 | 15 | 8·1 | 15 | 1005 | 15 | 67·5 | 15 | 37·5 | 15 |
| | C | 365 | 6 | 494 | 6 | 31 | 6 | 80 | 6 | 4·5 | 6 | 925 | 6 | 67·5 | 6 | 37·2 | 6 |
| *Miss R—, 57 yrs.*— Generalized M. Hodgkin; 25 mg. Nitrogen mustard in 6 days | A | 239 | 8 | 773 | 8 | 49 | 8 | 105 | 8 | 6·3 | 8 | 686 | 8 | 50·0 | 8 | 38·0 | 6 |
| | B | 403 | 13 | 738 | 13 | 48 | 13 | 149 | 13 | 6·9 | 13 | 565 | 13 | 44·3 | 13 | 36·9 | 13 |
| | C | 264 | 4 | 494 | 4 | 53 | 4 | 179 | 4 | 4·9 | 4 | 557 | 4 | 44·3 | 4 | 36·8 | 4 |
| *Mr. R—, 45 yrs.*— Generalized M. Hodgkin; 30 mg. Nitrogen mustard in 12 days | A | 457 | 4 | 695 | 4 | 32 | 4 | 198 | 4 | 8·0 | 4 | 1613 | 4 | 64·6 | 4 | 38·4 | 4 |
| | B | 548 | 18 | 580 | 18 | 35 | 18 | 220 | 18 | 6·2 | 18 | 1416 | 18 | 62·8 | 18 | 37·4 | 18 |
| | C | 414 | 8 | 461 | 8 | 34 | 8 | 199 | 8 | 4·3 | 8 | 1316 | 8 | 62·9 | 8 | 37·1 | 8 |
| *Mrs. N—, 57 yrs.*— Carc. metast. in neck and liver. Prim. tumour unknown; 25 mg. Nitrogen mustard in 5 days | A | 314 | 8 | 598 | 8 | 44 | 8 | 106 | 8 | 5·7 | 4 | 932 | 8 | 63·2 | 8 | 37·5 | 8 |
| | B | 383 | 12 | 659 | 12 | 44 | 12 | 80 | 12 | 8·1 | 12 | 899 | 12 | 62·0 | 12 | 37·6 | 12 |
| | C | 269 | 8 | 547 | 8 | 44 | 8 | 87 | 8 | 5·8 | 8 | 824 | 8 | 61·0 | 8 | 37·7 | 8 |
| *Miss S—, 55 yrs.*— Metastasized mammary carc.; 28 mg. Nitrogen mustard in 3 days | A | 313 | 4 | 419 | 4 | 51 | 4 | 23 | 4 | 3·3 | 4 | 1218 | 4 | 87·9 | 4 | 36·9 | 4 |
| | B | 377 | 13 | 527 | 13 | 51 | 13 | 33 | 13 | 6·1 | 13 | 1202 | 13 | 87·2 | 13 | 36·9 | 13 |
| | C | 321 | 4 | 444 | 4 | 48 | 4 | 30 | 4 | 4·8 | 4 | 1098 | 4 | 87·3 | 4 | 37·0 | 4 |
| *Mr. K—, 23 yrs.*— Reticulo sarcomatosis; 30 mg. Nitrogen mustard in 5 days | A | 207 | 3 | 1257 | 3 | 41 | 3 | 246 | 3 | 19·1 | 3 | 1600 | 3 | 70·0 | 3 | 37·6 | 3 |
| | B | 1473 | 10 | 2360 | 10 | 79 | 10 | 357 | 10 | 36·6 | 10 | 1600 | 10 | 64·9 | 10 | 37·9 | 10 |
| | C | 46 | 5 | 772 | 5 | 27 | 5 | 254 | 5 | 9·4 | 5 | 1435 | 5 | 64·0 | 5 | 37·4 | 5 |

A = Control period.

B = Metabolic changes during treatment.

C = Period of stabilization.

Two patients (1 M. Hodgkin and 1 metastasized thyroid carcinoma) received intravenous injections of triethylenemelamine. In Table II the metabolic changes are calculated in the same way as in Table I and in Fig. 3 the daily figures for the urinary excretions of metabolites, body weight and rectal temperature are depicted.

In the following paragraphs the same 3 relationships will be established as has been done in our former paper (Gerbrandy, Hellendoorn and Lokkerbol, 1958)

FIG. 2.—Changes in the urinary excretion of metabolites in a patient with generalized reticulosarcomatosis during treatment with nitrogen mustard.

about X-ray irradiation to 5 leukaemic and 3 cancer patients. For the sake of clarity the figures of our investigation on the effect of cytotoxic drugs will often be presented together with the corresponding figures of our irradiated patients.

(a) *The time-relationship*

During treatment with nitrogen mustard and triethylenemelamine the excretion of metabolites usually started to rise between the 2nd and 3rd day of the period of treatment. In Table III the first day of elevated excretion of phosphate and uric acid after commencing therapy is given in all our experiments with X-ray irradiation and cytotoxic drugs.

TABLE II.—*Urinary Excretion of Metabolites per 24 Hours Before, During and After Intravenous Injections of Triethylenemelamine (TEM)*

| Diagnosis and treatment | Period investigated | Phosphate | | Uric acid | | Potassium | | Calcium | | Urea | | Creatinine | | Body weight | | Rectal temp. | |
|---|---|---|---|---|---|---|---|---|---|---|---|---|---|---|---|---|---|
| | | (mg./day) | (No. of days) | (mg./day) | (No. of days) | (mM/day) | (No. of days) | (mg./day) | (No. of days) | (g./day) | (No. of days) | (mg./day) | (No. of days) | (kg.) | (No. of days) | (°C.) | (No. of days) |
| *Mrs. v. T—, 23 yrs.—* | A | 404 | 4 | 1129 | 4 | 44 | 4 | 346 | 4 | 10·0 | 4 | 958 | 4 | 49·5 | 4 | 39·3 | 4 |
| Generalized M. Hodgkin; | B | 739 | 11 | 1471 | 11 | 43 | 11 | 211 | 11 | 12·2 | 11 | 888 | 11 | 46·8 | 11 | 38·8 | 11 |
| 18 mg. TEM in 7 days | C | 554 | 6 | 954 | 6 | 45 | 6 | 186 | 6 | 5·1 | 6 | 729 | 6 | 45·9 | 6 | 37·2 | 6 |
| *Mrs. S—, 67 yrs.—* | A | 315 | 8 | 445 | 8 | 26 | 8 | 124 | 8 | 4·7 | 8 | 710 | 8 | 63·8 | 8 | 37·1 | 8 |
| Metastized thyroid carc.; | B | 408 | 11 | 602 | 11 | 27 | 11 | 147 | 11 | 7·0 | 11 | 703 | 11 | 62·8 | 11 | 37·0 | 11 |
| 18 mg. TEM in 5 days | C | 266 | 6 | 424 | 6 | 24 | 6 | 106 | 6 | 5·4 | 6 | 663 | 6 | 62·2 | 6 | 37·0 | 6 |

A = Control period.

B = Metabolic changes during treatment.

C = Period of stabilization.

Fig. 3.—Changes in the urinary excretion of metabolites in 2 patients during treatment by intravenous injections of TEM.

### Table III

| Name | Diagnosis | Therapy | First day of increased excretion after commencing therapy: Phosphate | Uric acid | Duration of increased excretion in days |
|---|---|---|---|---|---|
| Mr. de V— | Myel. leuk. | Rö | 2 | 2 | 29 |
| Mrs. M— | ,, | ,, | 1 | 1 | 27 |
| Mrs. Z— | Lymph. leuk. | ,, | 2 | 2 | 24+ |
| Mrs. v. D— | ,, | ,, | 2 | 3 | 41+ |
| Mr. P— | ,, | ,, | 3 | 3 | 19 |
| Mr. Gl— | Retic. sarc. | ,, | 3 | 4 | 16+ |
| Mr. A— | M. Hodgkin | $HN_2$ | 2 | 3 | 16+ |
| Mr. W— | ,, | ,, | 3 | 3 | 22+ |
| Miss B— | ,, | ,, | 3 | 2 | 15 |
| Miss R— | ,, | ,, | 2 | 2 | 13 |
| Mr. R— | ,, | ,, | 2 | ? | 18 |
| Mr. N— | Metast. carc. | ,, | 3 | 3 | 12 |
| Miss S— | ,, | ,, | 3 | 2 | 13 |
| Mr. K— | Retic. sarc. | ,, | 1 | 2 | 10 |
| Mrs. v. T— | M. Hodgkin | T.E.M. | 2 | 2 | 10 |
| Mrs. S— | Metast. carc. | ,, | 2 | 4 | 10 |

(b) *The total excess excretion of phosphate*

According to the findings of Waterhouse, Terepka and Sherman (1955) the P-content of 1 kg. of tumour tissue is assumed to be as follows : lymphatic leukaemia ± 4·7 g., myeloid leukaemia ± 3·2 g., M. Hodgkin ± 2·5 g., reticulosarcoma ± 3.2 g. and carcinoma ± 1·8 g. With these facts the total loss of tumour tissue

FIG. 4.—Correlation between calculated tumour loss (total excess excretion of phosphate) and fall of body weight in 18 patients treated with X-ray irradiation or cytotoxics.

is calculated from the excess excretion of phosphate. Because of the simultaneous rise of calcium excretion (skeletal breakdown) a correction of the phosphate excretion had to be made by means of the ratio Ca : P = 2·2 : 1. In Fig. 4 the calculated tumour loss from the excess excretion of P is compared with the fall of body weight in the same period. A significant correlation appeared to exist in which a calculated loss of 1 kg. of tumour tissue corresponded with a loss of at least 2 kg. of body weight.

TABLE IV

| | | | | | Palpable changes | | |
|---|---|---|---|---|---|---|---|
| Name | Diagnosis | Treatment | Excess excretion of P (g.) | Calculated loss of tumour (kg.) | Spleen (cm. below costal margin) | Liver (cm. below costal margin) | Lymph glands |
| Mr. A— | M. Hodgkin | $NH_2$ | 3·8 | 1·5 | 2 → 0 | 2 → 0 | + + + → + |
| Mr. W— | ,, | ,, | 2·9 | 1·1 | 2 → 0 | ½ → 0 | + + → + |
| Miss B— | ,, | ,, | 1·9 | 0·8 | 3 → 0 | Not palp. | + → ± |
| Miss R— | ,, | ,, | 1·9 | 0·8 | Not palp. | ,, | + + + → + |
| Mr. R— | ,, | ,, | 1·5 | 0·6 | ,, | 1 → 0 | + → 0 |
| Mr. N— | Metast. carc. | ,, | 1·0 | 0·6 | ,, | Not palp. | + → ± |
| Miss S— | ,, | ,, | 0·8 | 0·4 | ,, | ,, | + → + |
| Mr. K— | Retic. sarc. | ,, | 12·2 | 3·8 | (Rapid metastasis to all internal organs) | | |
| Mrs. V. T— | M. Hodgkin | TEM | 4·4 | 1·7 | 6 → 1 | Not palp. | + + + → + |
| Mrs. S— | Metast. carc. | ,, | 0·9 | 0·5 | 20 → 18 | 12 → 12 | + → + |

In Table IV the loss of tumour tissue calculated from the excess excretion of P is compared with other clinical data. The establishment of a quantitative relationship is impossible, of course, as not enough quantitative criteria of tumour loss are available. However, the decrease in the size of spleen and liver and the diminution of glandular swelling are a useful measure of therapeutic effect. It appears from the figures in Table IV that a certain correlation exists between the calculated tumour loss and the palpable tumour loss.

(c) *The mutual ratio between the excess excretion of metabolites*

In Table V the excess excretion of different metabolites in the urine during the period of metabolic changes is calculated in grams and millimols, taking the excretion in the control period as a base line. In 5 patients the mean rectal temperature during the control period was higher than 37·5° C. This was accompanied by an abnormally high excretion of urea and uric acid. In these cases we used the excretion level of the " period of stabilization " as a base for calculating the excess excretion of the different metabolites. The excess excretions of phosphate have been corrected for changes in the calcium excretion according to the ratio calcium : phosphorus = 2·2 : 1.

In Table VI the following ratios between the excess excreted metabolites are calculated : Mol P : Mol uric acid, Mol P : Mol K, Mol K : gram N (calculated from urea, uric acid and creatinine), Mol urea : Mol uric acid, gram N (calculated from urea, uric acid and creatinine) : gram P. In 3 cases the excess excretions of nitrogen calculated from the excess excreted amounts or urea, uric acid and creatinine were checked by a direct determination of the total nitrogen in the urine ; they differed no more than ± 10 per cent.

### DISCUSSION

The same relationship appeared principally to exist between the injection of nitrogen mustard or triethylenemelamine (TEM) and the excretion of metabolites as that between X-ray irradiation and the excretion of metabolites (Gerbrandy, Hellendoorn and Lokkerbol, 1958). This is concluded from the following facts :

1. A close time-relationship appeared to exist between the onset of the rise of metabolites and the commencement of injections (usually a difference of one day). It is not possible to correlate the end of the increase of the excretion of the metabolites with clinical data. It appears from the curves of the excretions of metabolites after injections of nitrogen mustard and TEM that the onset and rapidity of tumour destruction is practically the same for both substances, provided both are injected intravenously.

2. A close parallelism existed between the estimated tumour loss, calculated from the urinary excess excretion of phosphate, and the two approximate clinical standards of tumour loss, i.e. (*a*) the fall of body weight and (*b*) the diminishing of the size of an enlarged spleen, liver or lymph glands. As regards the fall of body weight this appeared to be twice as great as the tumour loss calculated from the excess excretions of phosphate. This will be caused by the loss of extracellular fluid and the loss of other tissues with a lower phosphate content. Furthermore the excess excretion of phosphate will not represent only loss of tumour tissue, as the injected cytotoxic drugs will also affect other rapid cell-dividing tissues as for instance bone marrow and such-like. Maybe the excess excretion of 0·8 g. phos-

TABLE V.—*The Urinary Excess Excretion of Metabolites During the Period of Metabolic Changes*

| Name | Diagnosis | Treatment | Phosphate (g.) | Phosphate (mMol) | Uric acid (g.) | Uric acid (mMol) | Potassium (mMol) | Urea (g.) | Urea (mMol) | Creatinine (mg.) | N (urea + uric ac. + creatin. (g.) | N total (g.) |
|---|---|---|---|---|---|---|---|---|---|---|---|---|
| Mr. A— | M. Hodgkin | $HN_2$ | 3,807 | 123·0 | 3,512 | 20·9 | 3·2 | 26·9 | 449 | −272 | 13·6 | — |
| Mr. W— | ,, | ,, | 2,867 | 92·5 | 3,388 | 20·2 | 48·4 | 23·3 | 388 | −1,912 | 11·3 | — |
| Miss B—* | ,, | ,, | 2,383 | 77·0 | 3,450 | 20·5 | 75·0 | 53·3 | 889 | +1,200* | 26·4 | — |
| Miss R—* | ,, | ,, | 1,991 | 64·2 | 3,186 | 19·0 | −29·5 | 25·7 | 429 | +98* | 13·1 | 12·3 |
| Mr. R—* | ,, | ,, | 2,240 | 72·3 | 2,142 | 12·8 | 49·6 | 33·7 | 526 | +1,800* | 17·1 | — |
| Mr. N— | Metast. carc. | ,, | 969 | 31·3 | 732 | 4·4 | 3·6 | 28·8 | 480 | −396 | 13·6 | — |
| Miss S— | ,, | ,, | 773 | 25·0 | 1,404 | 8·4 | −10·4 | 36·4 | 607 | −208 | 17·5 | — |
| Mr. K—* | Retic. sarc. | ,, | 13,802 | 445 | 15,880 | 94·7 | 519·0 | 271·7 | 4.520 | +1,650* | 132·5 | — |
| Mrs. v. T—* | M. Hodgkin | TEM | 2,073 | 66·0 | 5,687 | 33·6 | −27·5 | 78·0 | 1,299 | +1,749* | 38·9 | 34·2 |
| Mrs. S— | Metast. carc. | ,, | 908 | 29·3 | 1,727 | 10·3 | 7·7 | 24·8 | 412 | −77 | 12·1 | 13·4 |

* The period of metabolic changes is compared with the after period of stabilization because of fever in the control period. On account of the gradual fall of creatinine excretion a ± excretion of creatinine is noted in these cases.

TABLE VI.—*The Urinary Excess Excretion of Metabolites During the Period of Metabolic Changes*

| Name | Diagnosis | Treatment | Mol P : Mol uric acid | Mol P : Mol K | Mol K : Gram N {Urea, Uric ac., Creatin.} | Mol urea : Mol uric acid | Gram N {Urea, Uric ac., Creatin.} : Gram P |
|---|---|---|---|---|---|---|---|
| Mr. A— | M. Hodgkin | $HN_2$ | 5·9 | 40·7 | 0·2 | 21·5 | 3·6 |
| Mr. W— | ,, | ,, | 4·6 | 1·9 | 4·3 | 19·2 | 3·9 |
| Miss B— | ,, | ,, | 3·5 | 1·0 | 2·9 | 43·4 | 11·1 |
| Miss R— | ,, | ,, | 3·4 | —* | —* | 22·6 | 6·6 |
| Mr. R— | ,, | ,, | 5·7 | 1·5 | 2·9 | 43·9 | 7·6 |
| Mr. N— | Metast. carc. | ,, | 7·1 | 8·7 | 0·3 | 109·0 | 14·0 |
| Miss S— | ,, | ,, | 3·0 | —* | —* | 72·3 | 22·6 |
| Mr. K— | Retic. sarc. | ,, | 4·7 | 0·9 | 3·9 | 47·7 | 9·6 |
| Mrs. v. T— | M. Hodgkin | TEM | 2·0 | —* | —* | 38·7 | 18·8 |
| Mrs. S.— | Metast. carc. | ,, | 2·8 | 3·8 | 0·6 | 40·0 | 13·3 |

* Fall of potassium excretion during the period of metabolic changes.

phate in Miss S— with no perceptible clinical effect from the nitrogen mustard stems largely from the destruction of non-tumorous tissue.

3. A close parallel exists between the rise and fall of different metabolites such as phosphate, uric acid, urea and potassium.

4. The ratios between the excess excretion of the different metabolites did not differ fundamentally from the ratios found after X-ray irradiation. The differences between the individual experiments with cytotoxic drugs, however, were greater than between those with X-ray irradiation. This may be caused by the fact, that the cytotoxic drugs affected the whole body, and the X-rays mainly the tumour tissue. The closest agreement existed between the ratios Mol P : Mol uric acid in the experiments with irradiation and cytotoxic drugs ; in the irradiation experiments this ratio varied between 2·1 and 5·0 (mean 3·7) and in the experiments with cytotoxics the ratio varied between 2·0 and 7·1 (mean 4·3). These ratios are to be expected in breakdown of tumour tissue.

The ratio Mol P : Mol K has to be about 1 in tumour tissue. In our experiments with irradiation this ratio varied between 0·5 and 1·4 (mean 0·8). However, in only 4 of our 10 experiments with cytotoxics was this near 1 ; in 3 cases the ratio was much higher and in the other experiments the K-excretion had fallen during a rise of the other metabolites. It is possible that intravenous injections of cytotoxics affect the excretion of adrenal hormones and consequently the excretion of potassium. The variation in the ratio Mol K : gram N in the experiments with cytotoxics were too large to allow conclusions to be drawn.

In one respect the metabolic changes after injection of cytotoxic drugs differed from these after irradiation i.e. in the ratio gram N : gram P. According to Waterhouse, Terepka and Sherman (1955) this ratio amounts to about 15 in normal protoplasm but varies in leukaemic, sarcomatous and Hodgkin tissue usually between 5 and 15 and in carcinomatous tissue between 8 and 22. Our figures in the experiments with irradiation varied between 1·7 and 7·2 (mean 3·8) but in the experiments with cytotoxics this ratio varied between 3·6 and 23·6 (mean 11·1). The difference between the two sets of experiments has to be explained partly by the larger P-content of leukaemic cells than of Hodgkin and carcinomatous tissue and partly by the destruction of non-tumorous tissues with a relatively low P-content by the cytotoxic drugs.

In the experiments with cytotoxic drugs the same gradual fall of creatinine excretion and of serum creatinine was seen as in the irradiation experiments. We could not demonstrate a correlation between the fall of creatinine excretion and the fall of body weight. As contrasted with the irradiation experiments, an injection of cytotoxics was usually followed by a rise of calcium excretion (with the exception of Mrs. v. T— with M. Hodgkin who started with an elevated calcium excretion of 340 mg. a day). The cytotoxic drugs therefore usually also affect the bones to some extent.

As in the patients with chronic myeloid leukaemia, the excretion of uric acid in several patients with Hodgkin's disease was abnormally high during the control period. The phosphate excretion however, was normal during these periods. This reflects presumably the nearly total re-utilization of phosphate and the non- or only partial re-utilization of uric acid during an increased turnover of tumour cells. The excretion of both metabolites usually rises during the breakdown of tumour tissue but during the period of stabilization afterwards the excretion of uric acid falls below the control level and the phosphate excretion returns to about the control level. Assuming an inhibition of tumour growth by nitrogen mustard and TEM as in our irradiation experiments, the rise of excretion of phosphate and uric acid during the intravenous injections probably reflects the amount of metabolites originating from the normal metabolism of tumour cells that cannot be incorporated into new tumour cells. In this respect the case of Mr. K— with an extremely rapidly growing generalized reticulosarcomatosis seems to be interesting (Fig. 2). During the period of after-control the level of phosphate excretion fell for 3 days to zero but the excretion of uric acid stayed at an elevated level of about 700 mg. a day. Presumably all the phosphate available was required to be incorporated into the new tumour tissue, in contrast to the uric acid.

### SUMMARY

In 10 cancer patients (6 Hodgkin's disease, 3 metastasized carcinoma, 1 generalized reticulosarcoma), who were on a standardized low-protein diet, the urinary excretion of phosphate, uric acid, potassium, calcium, urea and creatinine was investigated during intravenous injections of nitrogen mustard (8 patients) and triethylenemelamine (2 patients). The effect of injections of cytotoxics on the excretions of metabolites appeared to be principally the same as of X-ray irradiation. This is concluded from the following facts :

1. A close time-relationship appeared to exist between the onset of the rise of metabolites and the commencement of injections (usually a difference of one day).

2. A close parallel existed between the estimated tumour loss, calculated from the urinary excess excretion of phosphate, and two approximate clinical standards of tumour loss, i.e. a fall of body weight and a diminishing of the size of an enlarged spleen, liver or lymph glands.

3. A close parallel existed between the rise and fall of the excretion of cell metabolites such as phosphate, uric acid, potassium and urea.

4. The ratios between the total excess excretions of phosphate and uric acid agreed with those found after X-ray irradiation and appeared to be in the range as could be expected from the destruction of tumour tissue. The excess excretions of potassium and nitrogen compared to phosphate, however, fell behind those after X-ray irradiation.

From the experiments with injections of cytotoxics as with X-ray irradiation a nearly total re-utilization of phosphate and a non- or only partial re-utilization of uric acid in growing tumour tissue were concluded.

### REFERENCES

ELIËL, L. P., PEARSON, O. H., KATZ, B. AND KRAINTZ, F. W.—(1950) *Fed. Proc.*, **9**, 168.

FENNINGER, L. D., WATERHOUSE, C. AND KENTMANN, E. H.—(1953) *Cancer*, **6**, 930.

GERBRANDY, J., HELLENDOORN, H. B. A. AND LOKKERBOL, H.—(1958) *Brit. J. Cancer*, **12**, 275.

HOMBURGER, F., BONNER, C. D. AND FISHMAN, W. H.—(1952) *Metabolism*, **1**, 435.

KRAKOFF, I. H.—(1957) 'The Leukamia'. New York (Acad. Press Inc.), p. 401.

SANDBERG, A. A., CARTWRIGHT, G. E. AND WINTROBE, M. M.—(1956) *Blood*, **11**, 154.

SPENCER, H., GREENBERG, J. AND LASZLO, D.—(1954) *Proc. Amer. Ass. Cancer Res.*, **1**, 16.

WATERHOUSE, C., TEREPKA, A. R. AND SHERMAN, C. D.—(1955) *Cancer Res.*, **15**, 544.